# Removal of dyes (BG, MG, and SA) from aqueous solution using a novel adsorbent macrocyclic compound

**DOI:** 10.1371/journal.pone.0275330

**Published:** 2022-10-06

**Authors:** Aveen F. Jalal, Nabil A. Fakhre

**Affiliations:** Department of Chemistry, College of Education, Salahaddin University-Erbil, Kurdistan Region, Iraq; Universiti Brunei Darussalam, BRUNEI DARUSSALAM

## Abstract

The use of macrocyclic compounds to remove organic dyes is fascinating because they have a wide surface area range and can be used for different things. new (14E, 34E)-7-Hydroxy-7, 8, 22, 23, 24, 25, 26, 27-Octahydro-6H, 16H, 33H Tetrabenzo[f,k,u,z][1,5,13,20]Tetraoxacycloheptacosine-16,33-Dione (HOTTD) was obtained by a simple high-dilution method, and characterized by FTIR, ^1^H-NMR, FESEM, EDX, and XRD. It worked well in removing organic dyes from aqueous solutions. Contact time, pH, dosage, initial concentration and temperature were studied. The optimum conditions were achieved by using 20 mg/L dye concentration, 50 mg dose of adsorbent and pH 9.0 at room temperature. The adsorption process was remarkably fast and reached equilibrium within 10 min for both Brilliant Green and Malachite Green while 70 min for Safranin. The batch adsorption experiments followed a pseudo 2^nd^ order and Langmuir model with maximum adsorption capacity 19.26 mg/g, 18.28 mg/g, and 14.35 mg/g for Brilliant Green, Malachite green and Safranin respectively. The process was endothermic and spontaneous in nature. Adsorbent regeneration test provides an excellent value 5 times.

## Introduction

Nowadays, a severe problem is the colored waters, especially the wastewaters from dyeing industries. Water pollution is still a significant global issue that distresses the worldwide human population [1]. Dyeing effluent is produced by numerous sectors such as leather, plastics, cosmetics, and textiles due to fast economic and industrial growth. Most dyes and their metabolites are hazardous to aquatic living species, posing a severe risk to the natural environment and human safety. As a result, dyes must be removed from the wastewater solution to an acceptable level [2,3]. Various techniques for removing these contaminants have been established, namely chemical precipitation, membrane separation, solvent extraction, and physical adsorption. Some of these methods have significant drawbacks, such as limited removal efficiency, high removal cost, and extended removal time [4].

Adsorption is a surface phenomenon in a multi-component fluid such as gas or liquid where solutes are attached to the surface of a solid substance by chemical and physical bonds [5]. Adsorbents can be usually classified as natural adsorbents, synthetic adsorbents, and semi-synthetic adsorbents [6,7]. Synthetic adsorbent such as polymeric resins, zeolites, or aluminosilicates; supramolecular macrocyclic compounds, magnetic nanoparticles (Fe_3_O_4_), and modified magnetic nanoparticles is the most popular adsorbent used by the researcher because of the high adsorption capacities [8,9]. Adsorption has the benefits of being simple to operate, efficient, and inexpensive compared to other approaches. Several efficient adsorbents have been produced in recent years because the adsorbent’s characteristics significantly impact its performance [4]. The adsorbent chosen in the adsorption process is essential; however, specific adsorbents have a high cost, poor recyclability, and limited capacity, limiting their application.

Macrocyclic compounds are defined as cyclic compounds with nine or more members (including all hetero atoms) and with three or more donor atoms [10]. Macrocyclic compounds including crown ethers, cyclodextrin, cucurbituril, calixarene, and columnarene. have always been the main body of supramolecular chemistry research [11]. Supramolecular macrocyclic compounds with macrocyclic cavities as host molecules have a strong adsorption effect on many guest molecules, and they have received extremely wide attention as dye adsorbents. Among many dye treatment technologies, adsorption shows unique advantages in the treatment of difficult-to-degrade dyes [11,12].

The cavity of the macrocyclic compound has a specific adsorption effect on many dyes, and the surface groups of the macrocyclic compound will also form strong non-covalent interactions with the dye, such as electrostatic interaction, π - π interaction, and hydrogen bonding. Their macrocyclic cavities can provide host-guest chemical effects, making them useful in separation and enrichment, molecular recognition, and supramolecular self-recognition. It has a high application value in assembly [12]. Because of the advantages of easy modification and inclusion effect, macrocyclic compounds have great application prospects in the adsorption and removal of dyes [13]. Macrocyclic compounds used as dye adsorbents can successfully achieve efficient and rapid dye adsorption and effectively promote the recycling of industrial wastewater [11]. When macrocyclic compounds are applied as dye adsorbents, they may accomplish efficient and quick dye adsorption while enhancing industrial wastewater recycling [14]. Many researchers have reported the use of supramolecular compounds for removal of organic dyes, Ting X. et.al. [2], And that reported in Hong-LiuJianget.al. And Rong G. et.al. [15,16].

The purpose of the paper is to prepare a new macrocycle compound [(14E, 34E)-7-hydroxy-7, 8, 22, 23, 24, 25, 26, 27-octahydro-6H, 16H, 33H-tetrabenzo[f,k,u,z][1,5,13,20]tetraoxacycloheptacosine-16,33-dione] using high-dilution method which is low cost, high product yield with less toxicity and highest chemical stability. The new crown compound have electron on the heteroatoms of the ring, which leads to increase the ability of the molecule with the ability to form complexes with the cations. The high-dilution principle, according to which low concentrations of the starting precursor favor cyclization over chain formation, was first developed by Paul Ruggli and Karl Ziegler for the cyclization of small organic molecules [17]. The prepared compounds were characterized by FTIR, ^1^H-NMR, FESEM, EDX, and XRD. The novel prepared crown ether is used as the adsorbent for the removal of dyes such as Brilliant Green, Malachite green, and Safranin. The adsorption kinetics, isotherms, thermodynamics, and regeneration of the adsorbent to remove dyes were studied in detail. Additionally, contact time, starting dye concentrations, pH, temperature, and adsorbent dosage were utilized for this study. The uptake of the adsorbent was applied in synthetic dyes sample.

## Material and methods

### Reagents

All reagents used were of analytical reagent grade stated. Brilliant Green (BG) (C_27_H_33_N_2_HO_4_S, M.wt = 482.65 g/mol) and Malachite Green (MG) (C_23_H_25_ClN_2_, M.wt = 365.0 g/mol) were purchased from Fluka. Safranin (SA) (C_20_H_19_N_4_Cl, M.wt = 350.84 g/mol) and Sodium carbonate (Na_2_CO_3_, ≥ 99.5%, M.wt = 105.99 g/mol) were purchased from BDH. Sodium hydroxide (NaOH, 97%, M.wt = 40.0 g/mol), potassium hydroxide (KOH, 85–100.5%, M.wt = 56.11 g/mol) were purchased from Scharlau. Hydrochloric acid (HCl, 37%, M.wt = 36.45 g/mol) was purchased from Merck. 1,3-dichloropropane (98%, M.wt = 128.98 g/mol) was purchased from Riedel-De Haen AG. Absolute Ethanol (C_2_H_5_OH, M.wt = 46.07 g/mol), Methanol (CH_3_OH, M.wt = 32.04 g/mol), 2-hydroxybenzaldehyde (C_7_H_6_O_2_, 98%, M.wt = 122.123 g/mol), 2-hydroxy acetophenone (C_8_H_8_O_2_, 99%, M.wt = 136.15 g/mol), and 1,6-dibromohexane (C₆H₁₂Br₂, 96%, M.wt = 243.96 g/mol) were purchased from Sigma-Aldrich.

### Preparation of new materials

According to the modified method [18], compound (1) [2,2’-((2-hydroxypropane-1,3-diyl) bis(oxy))dibenzaldehyde] was prepared by dissolving (9.77 g, 0.08 mol) of 2- hydroxybenzaldehyde in 90 mL ethanol. The solution was heated in a round bottle flask, and the compound was activated by adding anhydrous Na_2_CO_3_ (33.92 g, 0.32 mol). The 2-hydroxy1,3-dichloropropane (5.16 g, 0.04 mol) was added to the mixture and diluted to 40 mL of absolute ethanol. The mixture was refluxed by stirring for 8.0 hours. Then, the mixture was poured into an ice bath. The residue was filtrated, dried, and recrystallized using chloroform/methanol solution (1:1) to obtain the solid white product (yield 85%, m.p. 115–117°C).

Compound (2) [1,1-((hexane-1,6-diylbis(oxy))bis(2,1-phenylene))bis(ethan-1-one)] was prepared depending on the modified method [19], in which 2-hydroxy acetophenone (10.89 g, 0.08 mol) was dissolved in 100 mL absolute ethanol, heated and stirred on a magnetic stirrer. (33.92 g, 0.32 mol) of Na_2_CO_3_ was added slowly for activation of 2-hydroxy acetophenone. Then 1,6-dibromohexane (5.16 g, 0.04 mol) in ethanol (30 mL) was added dropwise. First, the mixture was refluxed for ten-hour at 180–200°C (the effect of the temperature was optimized). The mixture was put in an ice bath. The resulting precipitate was collected by filtration, dried, and recrystallized using chloroform/methanol solution (1:1) to give a white product (yield 82%).

Compound (3) **[**(14E, 34E)-7-Hydroxy-7, 8, 22, 23, 24, 25, 26, 27-Octahydro-6H, 16H, 33H Tetrabenzo[f,k,u,z][1,5,13,20]Tetraoxacycloheptacosine-16,33-Dione (HOTTD)] as macrocyclic compound (MC) was prepared according to the modified method [20], A mixture of compound (1) (75 mg; 0.25 mmol) and compound (2) (88.6 mg; 0.25 mmol) was dissolved in a KOH solution (10%, 130–160 ml) in MeOH/H_2_O (3:1) and the mixture was refluxed for 6.0 h. The reaction mixture was left at room temperature with stirring for five days; then, the solvent was reduced to nearly half volume under reduced pressure. The resulting precipitate was collected by filtration, dried, and recrystallized using chloroform/methanol solution (1:1) to obtain yellow precipitation (yield 82%).

### Characterization of prepared materials

The synthesized macrocyclic compound was characterized in a way that covers a large area. Fourier transfer infrared spectra were carried out (Shimadzu IRAffinity-1 Fourier Transform Infrared (FTIR) spectrometer). The morphologies of particles were observed using FESEM coupled with EDX, (TESCAN MIRA3 FEG-SEM, Czech Republic at 15kV under a low vacuum after coating with a gold thin film, with an SE detector for EDX), and The powder XRD patterns were recorded by PANalytical X’Pert Pro diffractometer with Cu-kα radiation (1.5406 Å, 45 kV, 40 mA) and a 2θ between 5 to 80°. Finally, 1H-NMR (broker AVANCENEO (400MHz) spectrometer).

### Batch adsorption procedure

In this investigation, all experiments of adsorption were examined, including dosage effect using various amounts of sorbent 25–100 mg, pH effect from 2.0–11.0, the impact of initial dyes concentration 5–150 mg/L, temperature effect from 5–50°C and contact time 0–24 h.

Batch adsorption process applied with 50 mg of adsorbent and 10 mL of 20 mg/L of dye solution at room temperature and pH 9.0. Except for the adsorption kinetics experiment, the tube was kept in a water bath oscillator thermostat for 24 h at 200 rpm. The centrifuge was used to separate the sorbent and sorbate when the adsorption was completed at the specified time. After the adsorption process, the concentration of BG, MG, and SA dyes was determined using a Spectrophotometer at 618, 625, and 520 nm, respectively. The quantity of BG, MG, and SA sorbed onto sorbent and removal percentage were computed according to the following formula Eqs 1 and 2:

$$q_{e}=\frac{(C_{i}‐C_{e})⨯V}{m}$$ (1)

$$R\%=\frac{\left(C_{i}‐C_{e}\right)}{C_{i}}⨯100$$ (2)

Where equilibrium capacity of a sorbent is represented by qe (mg/g), V (L) is a volume of solution, m (g) means sorbent mass, both C_i_ and C_e_ (mg/L) were initial, and equilibrium BG, MG, and SA concentration, respectively and R was the removal percent of dyes.

Isotherms are mathematical equations that describe the adsorption behavior of a particular adsorbent-adsorbate combination. Langmuir, Freundlich, and Temkin models were used to agree with the adsorption data to investigate the mechanism of the adsorption process [21,22]. The linear version of the Langmuir equation depicts in Eq 3.


$$\frac{C_{e}}{q_{e}}=\frac{1}{qm\;KL}+\frac{C_{e}}{qm}$$ (3)

K_L_ is a Langmuir constant related to adsorption capacity (mg/g), qm. That can be connected with variations in the appropriate area and porosity of the adsorbent, implying that adsorption capacity will be more significant if the surface area and volume are large. The separation factor R_L_ is a dimensionless constant that expresses in Eq 4 the essential characteristics of the Langmuir isotherm.


$$RL=\frac{1}{1+KL\;C_{e}}$$ (4)

R_L_ values indicate the adsorption to be unfavorable when R_L_> 1, linear when R_L_ = 1, favorable when 0 < R_L_ > 1, and irreversible when R_L_ = 0.

While the following Eqs 5 and 6 represent Freundlich and Timken isotherms linear forms:

$$log\;q_{e}=log\;KF+\frac{1}{n}logC_{e}$$ (5)

$$q_{e}=at+2.303bt\;logC_{e}$$ (6)

1/n indicates adsorption capacity, while the K_F_ represents adsorption capacity (L/mg). The Temkin constant bt is related to the heat of sorption (J/mol) and the Temkin isotherm constant at (L/g). Thermodynamic parameters such as ΔG°, ΔH°, and ΔS° were calculated using Eqs 7–9.


$$Kd=\frac{q_{e}}{C_{e}}$$ (7)

$$lnKd=\frac{\Delta S{}^{\circ}}{R}-\frac{\Delta H{}^{\circ}}{RT}$$ (8)

ΔG˚=‐RTlnKd (9)

Where T is the absolute temperature K; R is the universal gas constant 8.314 J/mol. K; K_d_ is the adsorption distribution constant derived using qe/Ce and qe mg/g [23].

This research investigated the adsorption kinetics by fitting the experimental data to pseudo 1^st^ order, pseudo 2^nd^ order, and intra-particle diffusion kinetic models developed in the previous literature. Then, using linearized pseudo 1^st^ order, pseudo 2^nd^ order, and intra-particle diffusion, represent them in the following Eqs 10–12, [24].


$$log(q_{e}-qt)=log\;q_{e}-\frac{k_{1}t}{2.303}$$ (10)

$$\frac{t}{qt}=\frac{1}{q_{e}}+\frac{1}{k_{2}\;q_{e}{}^{2}}$$ (11)

$$qt=k_{3}\;t^{0.5}+C$$ (12)

The k_1_ (min^-1^) and k_2_ (g/mg.min) are connected to the pseudo 1^st^ and 2^nd^ order kinetic models. The intercept and the slope of the plot of log(q_e_- qt) against t were used to determine the value of qe. k₃ is the intra-particle diffusion rate constant (mg/g.min^1/2^) which can be obtained from the slope of the straight line and C is an intra-particle diffusion rate constant related to the boundary layer thickness (mg/g) which can be obtained from the intercept of the straight line [25].

The adsorption/desorption study was cared by 50 mg of MC placed in a conical flask containing dyes solution 10 mL, 20 mg/L and the mixture was shaken in a water bath using thermostat shaker for 70 min at room temperature. The mixture was separated by centrifuge and the final dye concentration was found. The adsorbent was recycled by washing with ethanol and water under constant stirring for three times (1.0 h), respectively. The experiment repeated and dipped the adsorbent into another dye solution to begin a new adsorption process. The recyclability of the material was followed up to five consecutive adsorption-desorption cycles.

## Results and discussion

### Characterization of new adsorbent

A new crown ether was prepared by condensation reaction between the aldehyde and ketone.

### FT-IR

The new aldehyde (2, 2-(propane-1,3-diylbis(oxy) dibenzaldehyde) (Compound 1) was prepared in good yield (85%) based on Williamson ether synthesis between 2-hydroxy Benzaldehyde and 1,3 dichloro-2-propanol [18].




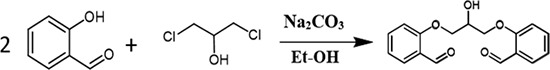




**Fig 1:**
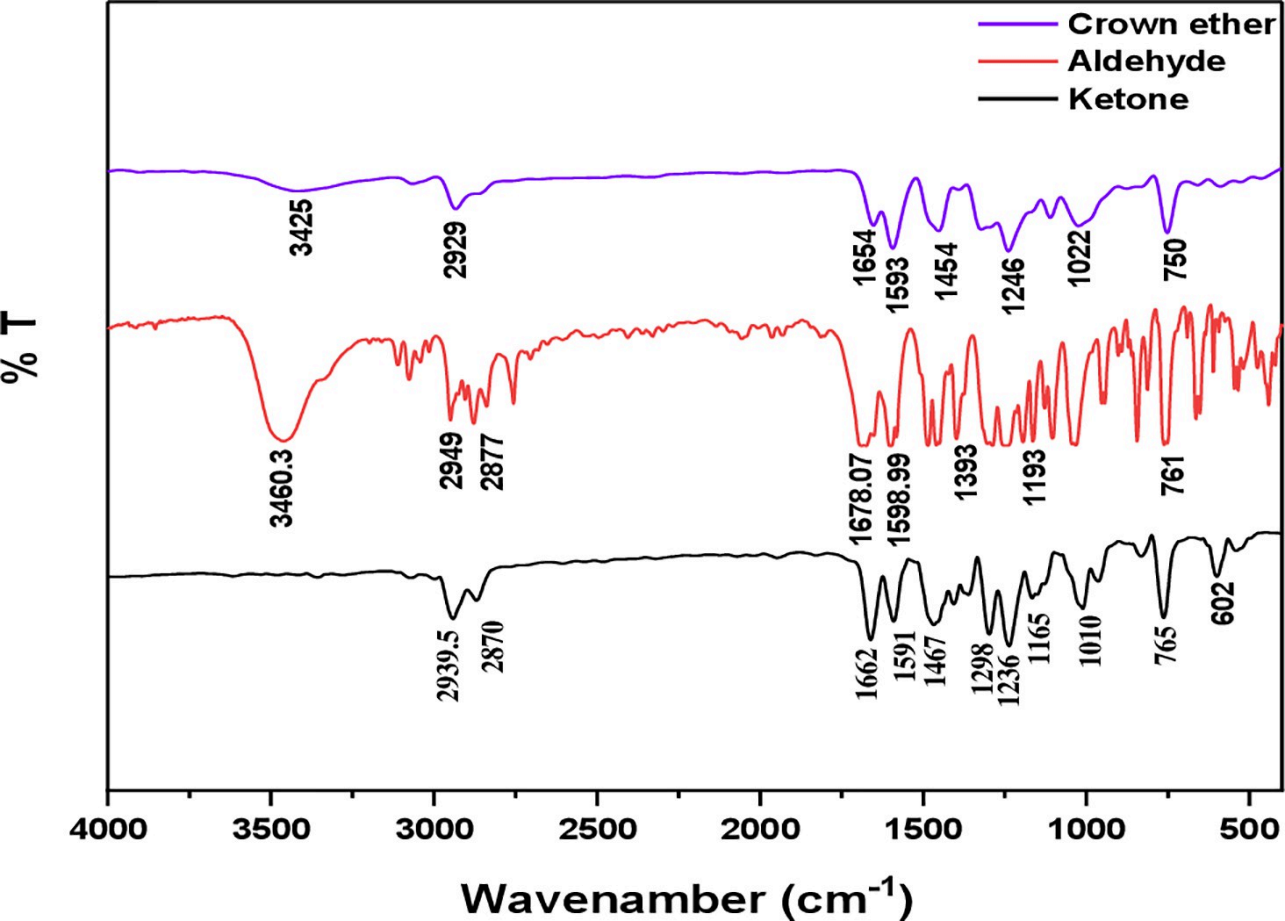
FT-IR spectrum of Crown ether, Aldehyde, and Ketone.

The structure of the synthesized compound was determined using FT-IR techniques, FT-IR spectrum of aldehyde compound (1) (**Fig 1**), the appearance of broadband at 3460.3 cm^-1^ is attributed to the OH group, and the strong band at 1678 cm^-1^ refers to the carbonyl group while 2877, 2949 cm^-1^ C-H Aliphatic.

A new ketone 1,1-((hexane-1,6-diylbis(oxy))bis(2,1-phenylene))bis(ethan-1-one) compound (2) was prepared in good yield (82%) based on Williamson ether synthesis between 2-hydroxy acetophenone and 1,6-dibromohexane.




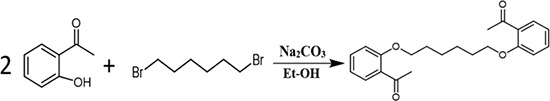




FT-IR characterized the structure of the new synthesized compound (2) (**Fig 1**), the appearance of a strong band at 1662 cm^-1^ refers to the carbonyl group of the ketone, while 2870, 2939.5 cm^-1^ refers to C-H aliphatic.

According to the reported procedure [20]. A new crown ether was synthesized by modifying Classen–Schmidt condensation between 2,2’-((2-hydroxypropane-1,3-diyl) bis(oxy))dibenzaldehyde (Compound 1) and 1,1-((hexane-1,6-diylbis(oxy))bis(2,1-phenylene))bis(ethan-1-one) in a basic medium was synthesized in good yield (82%).




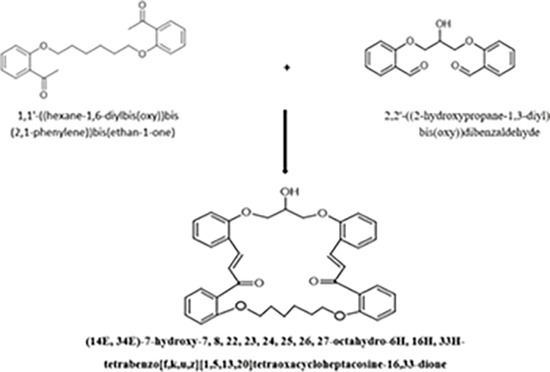




FT-IR characterized of a new synthesized crown ether compound (3) structure, as shown in **Fig 1**. FT-IR spectrum of compound shows shifting of the carbonyl group to the lower value 1678 and 1662 cm^-1^ to the 1654 cm^-1^ corresponds to the carbonyl group of α,β-unsaturated ketone, that is a useful guide to identify conjugated enone as described in the literature.

#### ^1^H-NMR

The ^1^H-NMR spectrum of compound (1) (**S1 Fig**), shows a signal at 4.26 ppm refers to the hydroxyl group, while 4.31 ppm belongs to methylene proton, a quintet at 4.55 ppm refers to CH- aliphatic protons, a multiple at 7.0–7.8 ppm corresponds to the eight protons of two aromatic ring, and a single signal at 10.48 ppm belongs to the proton of an aldehyde group.

The ^1^H-NMR spectrum of compound (2) (**S2 Fig**), as illustrated by a signal at 2.65 ppm, refers to (s, 6H, 2 × -COCH3), 4.10 ppm belonging to methylene proton, a multiple at 1.62–1.92 ppm refers to eight CH- aliphatic protons and multiples at 6.95–7.03 ppm corresponds to the eight protons of two aromatic ring.

The ^1^H-NMR spectrum of compound (3) (**S3 Fig**) shows the disappearance of an aldehyde group at 10.48 ppm and the appearance of multiple signals in the range 0.83–1.3 ppm and 2.5 ppm, which refers to eight CH-aliphatic, multiple protons in the range 3.47–4.02 ppm refers to nine protons of (4X-OCH_2_-, and X-CH-OH), a signal at 5.48 ppm refers to X–OH, and a multiple at 6.91–7.81 ppm fits 16 protons of four aromatic ring and 4 protons of α,β unsaturated enones.

#### FESEM

**Fig 2:**
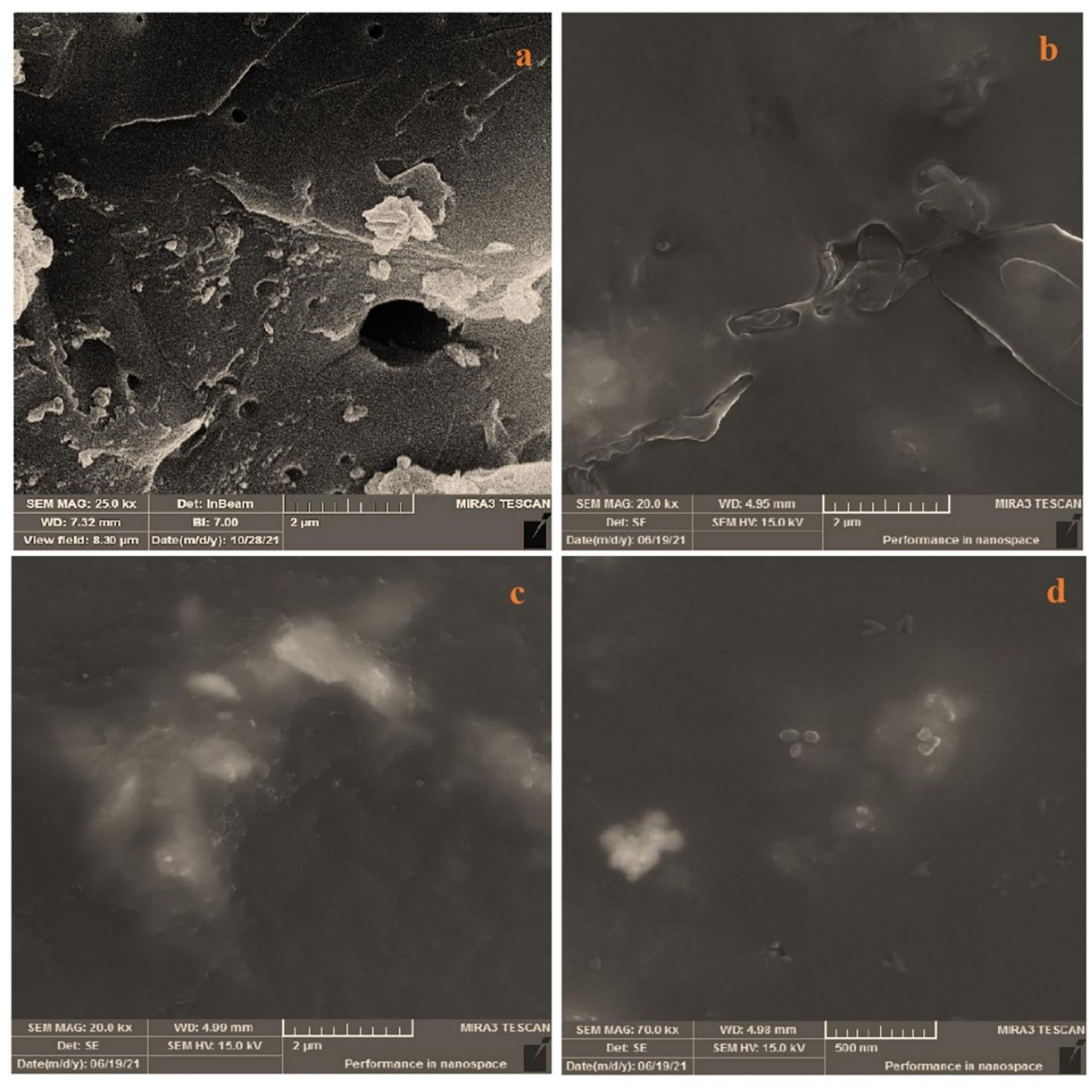
FESEM images of (a) MC before adsorption of dye (magnification: 25 kx, 2 μm), (b) MC after adsorption of BG (magnification: 25 kx, 2 μm), (c) MC after adsorption of MG (magnification: 25 kx, 2 μm), and (d) MC after adsorption of SA (magnification: 25 kx, 500 nm).

FESEM (Field Emission Scanning Electron Microscopy) has been a primary tool for characterizing the surface morphology and fundamental physical properties of the adsorbent before and after the adsorption of dyes. Fig 2(A) Shows many pores in the adsorbent surface with various grooves favorable for adsorption. A significant change is observed after dyes adsorption Fig 2(B)–2(D) in the adsorbent structure. The adsorbent surface was changed, and dye molecules coated on all the surfaces and pores of the adsorbent decreased after adsorption. This surface property should be considered a factor for dye binding.

#### EDX

**Fig 3:**
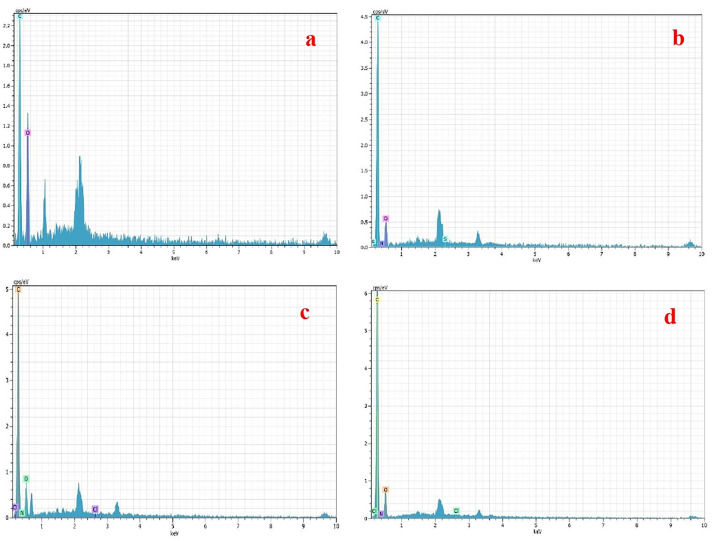
EDX of MC (a), MC after adsorption of BG (b), MC after adsorption of MG (c), and MC after adsorption of SA (d).

The EDX analysis indicated the presence of certain components in the composition, which were then determined using the spectrum. As illustrated in **Fig 3(A)**, C and O are the two most essential components in producing macrocyclic compounds. In **Fig 3(B)–3(D)** appearing new atoms such as N, S, and Cl, the dye molecules successfully sorption on the sorbent.

#### XRD

**Fig 4:**
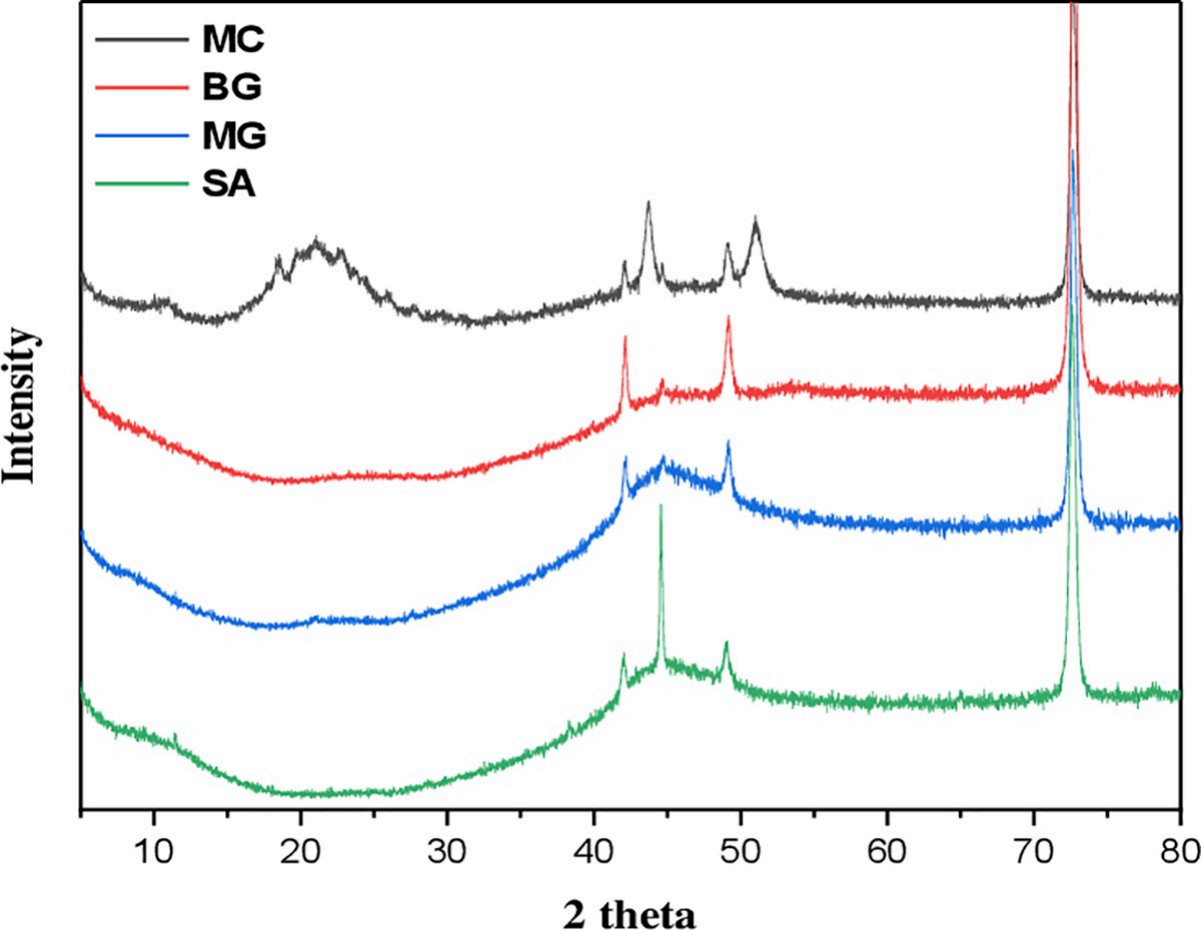
Demonstrates the X-ray diffraction spectra for MC (black line), MC after adsorption of BG (red line), MC after adsorption of MG (blue line), and MC after adsorption of SA (green line).

The XRD analysis was completed, and the findings are shown to examine the crystallographic structure of the adsorbent. XRD patterns were presented in **Fig 4**. When comparing the XRD pattern of MC before adsorption and after adsorption of BG, MG, and SA dyes. XRD pattern of MC has broad peaks (~22°or ~25°), demonstrating the semicrystalline or amorphous structure of macrocyclic compounds [26]. After the adsorption of dyes, it was discovered that they significantly changed with a decrease in the crystalline structure. Consequently, dye molecules preferentially adsorb via chemisorption and only partly through physisorption. While XRD analysis shows no significant changes in peak locations 2θ >30, only an increase in intensity due to dyes adsorption of adsorbent, this indicates that the crystal structure of the adsorbent was not destroyed after adsorption of dyes.

### Adsorption time and kinetic study

**Fig 5:**
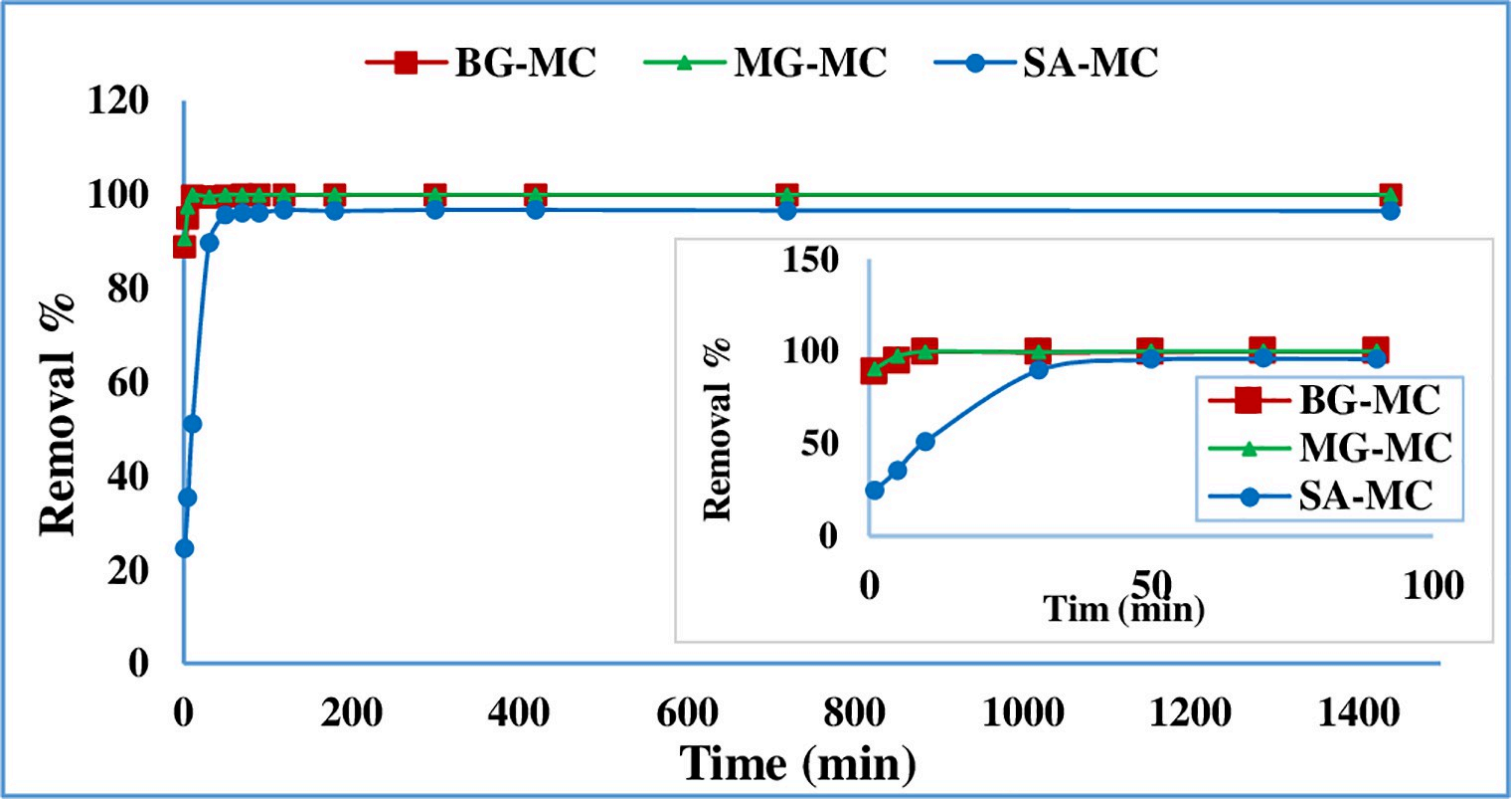
Adsorption time impact of BG, MG, and SA removal onto MC.

The impact of contact time on the removal percentage of BG, MG, and SA was achieved over the time interval of 0–24 h under fixed conditions values (pH = 9, adsorbent dosage 50mg/10mL and 20 mg/L concentration of dye), the results are shown in **Fig 5**.

From the chart, we can see in the initial minutes, a removal percent value of about 89.00% for BG, 90.50% for MG, and 24.60% for SA was attained, when the contact time reached 10 min for both BG and MG, and 70 min for SA, the value of removal increase to 99.66% and 99.86%, and 96.19% of BG, MG, and SA respectively. By further increasing the contact time after 1.0 min, the removal percentage was increased slightly reaching the maximum value at 10 min, after this time there was no significant change by increasing the time. The optimized equilibrium time is about 10 min for BG and MG, enough for further experiments.

While for SA, According to the diagram, the removal percent increased rapidly when contact time reached 70 min, then increased slightly, reaching a maximum value of about 96.19%. Thus, the subsequent adsorption experiments were performed at 70 min.

The result showed that the prepared adsorbent has short equilibrium time which shows the adsorbent have enough number of active site for a given concentration. Compared with the literature, this equilibrium time is better than the ones previously reported by Ahmed et al. [27] and Abbas [28] for BG, Palapa et al. [29] and Alorabi [30] for MG and Abukhadra and Mohamed [31] and Mullerova et al. [32] for SA.

The kinetic studies can give information about the nature of the adsorption system (chemical or physical) and the strength of held between the adsorbate and the used adsorbent [31]. Pseudo-first-order, pseudo-second-order, and intra-particle diffusion kinetic models were employed to match the experimental sorption data in this work, **S4 Fig** and **Table 1** provide the fitting parameters for the kinetic models. When Pseudo-first-order is compared to the pseudo-second-order kinetic model, the R^2^ value of the pseudo-second-order kinetic model is more significant for BG, MG, and SA; the value is 1.0, 0.9996 and 1.0 respectively, suggesting that the adsorption of all dyes onto crown ether adsorbent occurred by the chemisorption mechanism. Moreover, the qe, exp value derived by pseudo-second-order was consistent with the qe, cal. value, supporting the pseudo-second-order kinetic model. Similar trends have been reported by other adsorbents [28, 33] for BG, [30,34] for MG and [32]for SA.

**Table 1:** Kinetic characteristics for the adsorption of BG, MG, and SA.

Kinetic models	Parameter	BG	MG	SA
	q_e_ (exp.) (mg/g)	4.0	4.0	3.869
Pseudo-first order	q_cal_ (mg/g)	0.1803	0.1881	1.622
	k_1_ (min^-1^)	1.714	0.1096	0.0553
	R^2^	0.6622	0.7443	0.8433
Pseudo-second order	q_cal_ (mg/g)	4.0	4.001	3.869
	k_2_ (g/mg.min)	3.27	2.715	0.1637
	R^2^	1.0	0.9996	1.0
Intra-particle diffusion	C (mg/g)	3.651	3.572	0.2389
	k_3_ (mg/g.min^½^)	0.0609	0.0612	0.596
	R^2^	0.9907	0.9832	0.9854

The intra-particle diffusion explains the movement of dye molecules from the bulk solution phase to the solid phase. The constants k₃ and C are calculated and listed in **Table 1**. As shown in **S4 Fig** the plots of qt vs. t ^1/2^ for BG, MG, and SA dye molecules are shown in two stages. The first straight portion illustrates macropore diffusion and the second stage describes micropore diffusion. All the intra-particle diffusion rate constants C in this work are not zero which implies that the adsorption method may not be predominantly controlled by intra-particle diffusion [35].

### Effect of adsorption conditions

#### Effect of pH on adsorption

**Fig 6:**
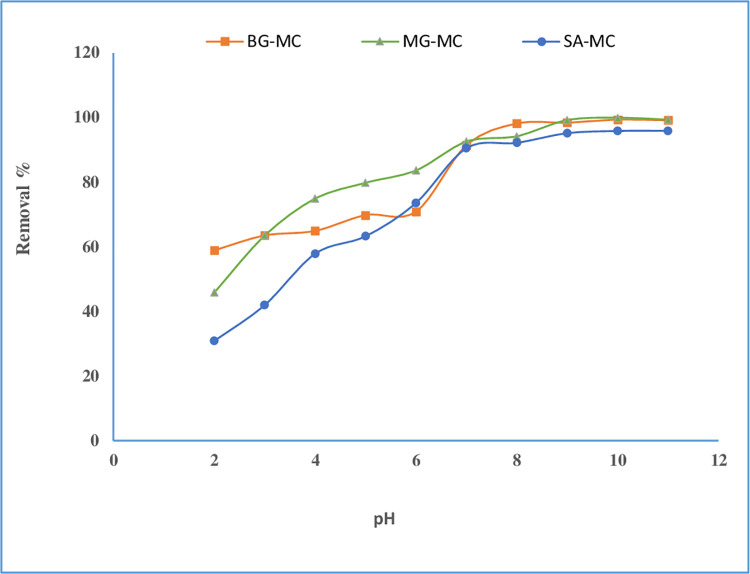
Effect of pH on removal percentage of BG, MG, and SA onto adsorbent.

A significant factor in the adsorption experiment is the pH solution. The dye becomes more unstable at a particular pH and becomes easier to remove from the aqueous solution via selective sorbent. pH has a significant impact on the ionization and charge of the functional group of the adsorbate [36]. To look at the effects of solution pH on the adsorption of BG, MG, and SA dyes, the pH of the adsorbate solution was changed from 2.0 to 11.0 and initially adapted with either HCl or NaOH 1.0 M and 0.1 M. The experiment was performed at a fixed amount of adsorbent 50 mg/10 mL, an initial dye concentration of 20 mg/L, and under a contact time of 70 min. **Fig 6** illustrates the removal efficiency of BG increase 58.9% - 96.46% with an increasing pH range of 2.0–9.0, then the removal efficiency constant until it reaches pH 11.0. At the same time, the removal percent for MG 45.9% - 99.19% was observed in pH 2.0–9.0. After that, adsorption is almost constant until it reaches pH 11.0. The removal percent for SA 30.9% - 95.88% was observed in pH 2.0–10.0; then, it was constant. pH 9 was chosen as the optimum pH for the removal of dyes onto the adsorbent. Similar findings have been reported in other publications for BG [37], MG [38], and SA [39] dyes.

#### Concentration of initial dyes effect

**Fig 7:**
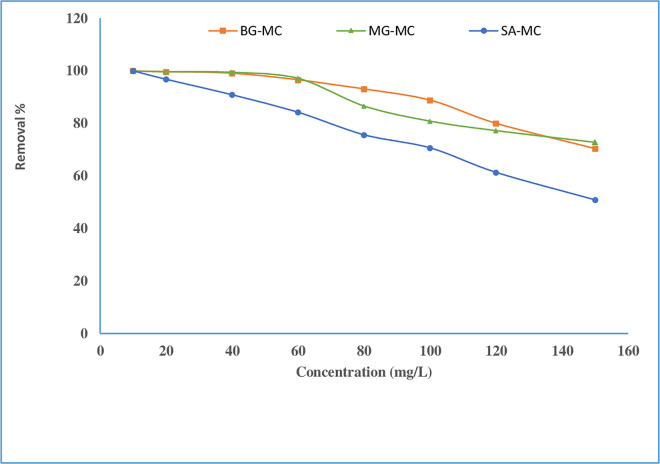
Effect of concentration on adsorption of BG, MG, and SA onto adsorbent.

The impact of initial dye concentrations ranging from 5.0 mg/L to 150 mg/L and under the optimized values of the other experimental conditions (adsorbent dosage of 50 mg/10 mL, initial pH of 9, and contact time of 70 min) on removal percent was studied to assess the adsorption performance of dye molecules on the adsorbent. As presented in **Fig 7**, raising the starting dye concentration reduced the adsorption percent. As a result, when the initial concentration rises from 10 mg/L to 60 mg/L for BG, MG, and SA, the removal percent between 99.99% - 96.61%, 99.96% - 97.13%, and 100% - 84.22% respectively, but when the concentration rises beyond 60 mg/L, the percentage drops slightly. This behavior may be described in the following way, the degree of active sites was sufficient to occupy dye molecules at low concentrations, resulting in high removal efficiency. Conversely, with higher starting dye concentrations and the same adsorbent mass, a scarcity of adsorption sites may develop, resulting in a decrease in removal effectiveness due to the active site of adsorbent saturated by dye molecules at extremely high starting concentrations [40,41].

#### Adsorbent dose effect

**Fig 8:**
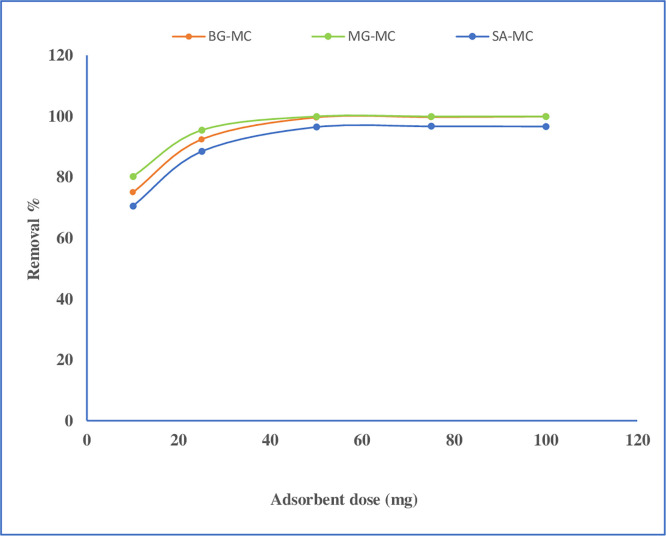
Effect of adsorbent dose on dye removal.

On dye molecule sorption, the action of adsorbent weight was studied. As observed in **Fig 8**, the optimal weight for adsorbent was 50 mg, which resulted in 99.68%, 100%, and 96.5% removal for BG, MG, and SA, respectively. Initially, the adsorption process increases as the adsorbent mass increases, but after an optimal dose is achieved, it stays constant. Due to a more significant number of active sites, clearance efficiency is anticipated to rise. Any further addition of the adsorbent seemed to have no significant effect on adsorption, which might be due to adsorption site overlapping due to adsorbent particle crowding [42,43]. Generally, 50 mg/10mL was taken as an optimum quantity for this work.

#### Effect of temperature

**Fig 9:**
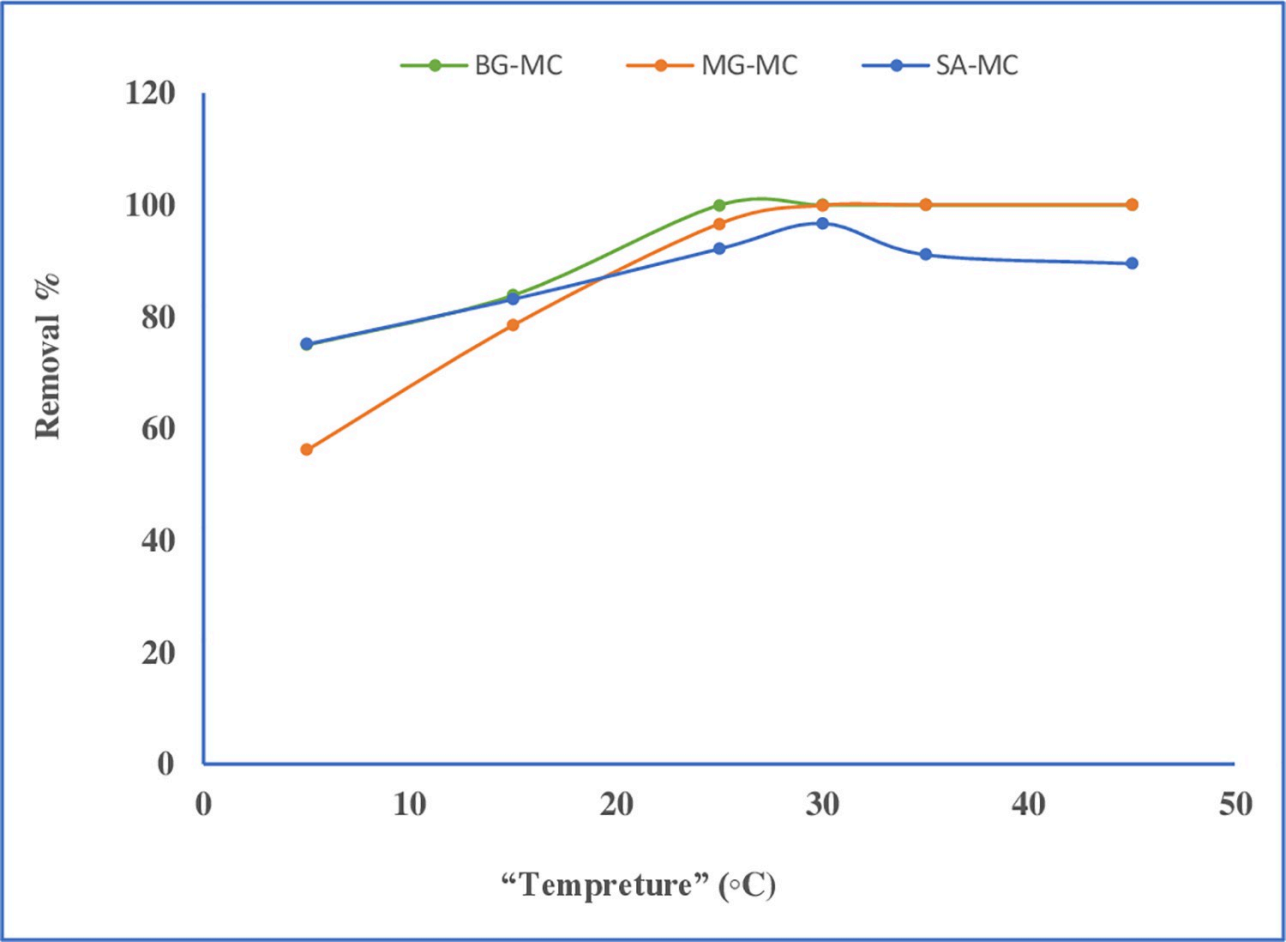
Effect temperature on dye removal percent.

The mobility of molecules and ions in a solution is affected by temperature. This can include ion adsorption since ions must be mobile to ’collide/interact’ with the adsorbent and enhance adsorption, which is especially important in batch adsorption investigations. Therefore, the effect of temperature on the removal % of dyes was studied, and the results are shown in **Fig 9**. The measurements were carried out at varying temperatures ranging from 5.0 to 45°C. As observed from the figure, the removal % increased by increasing temperature from 5.0 to 25°C for BG and 5.0 to 30°C for both MG and SA. Then removal % remained constant for BG and MG, above 30°C for SA. There is an apparent decrease in the removal percentage. As a result, throughout the rest of this study, the room temperature was given maximum removal, and it was selected as the optimum removal temperature for all dyes [39,44,45].

According to basic thermodynamic principles, energy cannot be acquired or lost in an isolated system, and entropy change is the only driving factor. Therefore, environmental engineers must examine both energy and entropy factors to determine which process will occur naturally. The effect of temperature on the BG, MG, and SA dyes’ adsorption onto sorbent was investigated using thermodynamics. Kd, the thermodynamic equilibrium constant, may be used to calculate the thermodynamic parameters Eq (7). The Eqs (8) and (9) are used to compute the standard Gibbs free energy ΔG° kJ/mol, standard enthalpy change ΔH° kJ/mol, and standard entropy change ΔS° J/mol K.

The slope and intercept of the linear plot of ln Kd versus 1/T were used to get the values of ΔH and ΔS, whereas the values of ΔG were determined using the equation above. As may be seen, the adsorption of dyes is more favorable at higher temperatures, implying that the adsorption process is endothermic, as shown by the positive values of ΔH **(Table 2)**. The results suggest that chemisorption might be dominant. Following that, a positive value of ΔS implies a high degree of randomness at the interface between the solid and the solution. The negative values of ΔG indicate that the adsorption of all dyes onto the adsorbent occurred as a result of spontaneous adsorption [46,47].

**Table 2:** Thermodynamic parameters for the adsorption of BG, MG, and SA dye molecules onto the adsorbent.

Dyes	T(K)	ΔG (kJ/mol)	ΔH(kJ/mol)	ΔS (J/mol K)
**BG-MC**	278	+1.183	+ 153.38	+ 564.89
	288	-0.0893		
	298	-13.998		
	303	-15.09		
	308	-15.90		
	318	-18.24		
**MG-MC**	278	+3.143	+166.72	+585.56
	288	-1.032		
	298	-4.303		
	303	-13.344		
	308	-16.65		
	318	-18.26		
**SA-MC**	278	+1.160	+48.45	+170.2
	288	+0.0244		
	298	-2.116		
	303	-4.454		
	308	-4.545		
	318	-4.783		

#### Adsorption isotherms models study

The equilibrium of sorption is an essential physicochemical feature in explaining adsorption behavior in solid-liquid systems. The object of adsorption isotherms is to establish a relationship between the adsorbate concentration in bulk and the quantity adsorbed at the interface [46]. The results were analyzed using the Langmuir, Freundlich, and Temkin isotherms. The parameters are determined and presented in **Table 3**. The applicability of the isotherm to describe the adsorption process was judged by the value of correlation coefficient R^2^. According to the results, the adsorption of all dyes is best fitted with the Langmuir isotherm. It is concluded that reactive zones on the adsorbent sites are homogenously covered by dyes molecule. R_L_, separation factor is favorable for adsorbent toward BG, MG, and SA with ranges (0.0244–0.0020), (0.0316–0.0027), and (0.0455–0.0041), respectively, within the concentration range of 10 to 120 mg/L, pH = 9.0, adsorbent dosage 50 mg/10 mL and room temperature.

**Table 3:** The parameters of the Langmuir and Freundlich and Temkin models for dye adsorption onto adsorbent.

Isotherm models	Parameter	BG	MG	SA
**Langmuir**	q_m_ (mg/g)	19.26	18.28	14.35
	K_L_ (min^-1^)	3.99	3.055	2.010
	R^2^	0.9992	0.9994	0.9991
	R_L_	(0.0244–0.0020)	(0.0316–0.0027)	(0.0455–0.0041)
**Freundlich**	1/n	0.2282	0.2656	0.3633
	K_f_	9.166	8.218	4.304
	R^2^	0.9807	0.9308	0.9618
**Temkin**	a_t_	11.467	10.381	5.426
	b_t_	1.74	2.0570	2.438
	R^2^	0.9118	0.9781	0.9949

#### Regeneration and reusability

**Fig 10:**
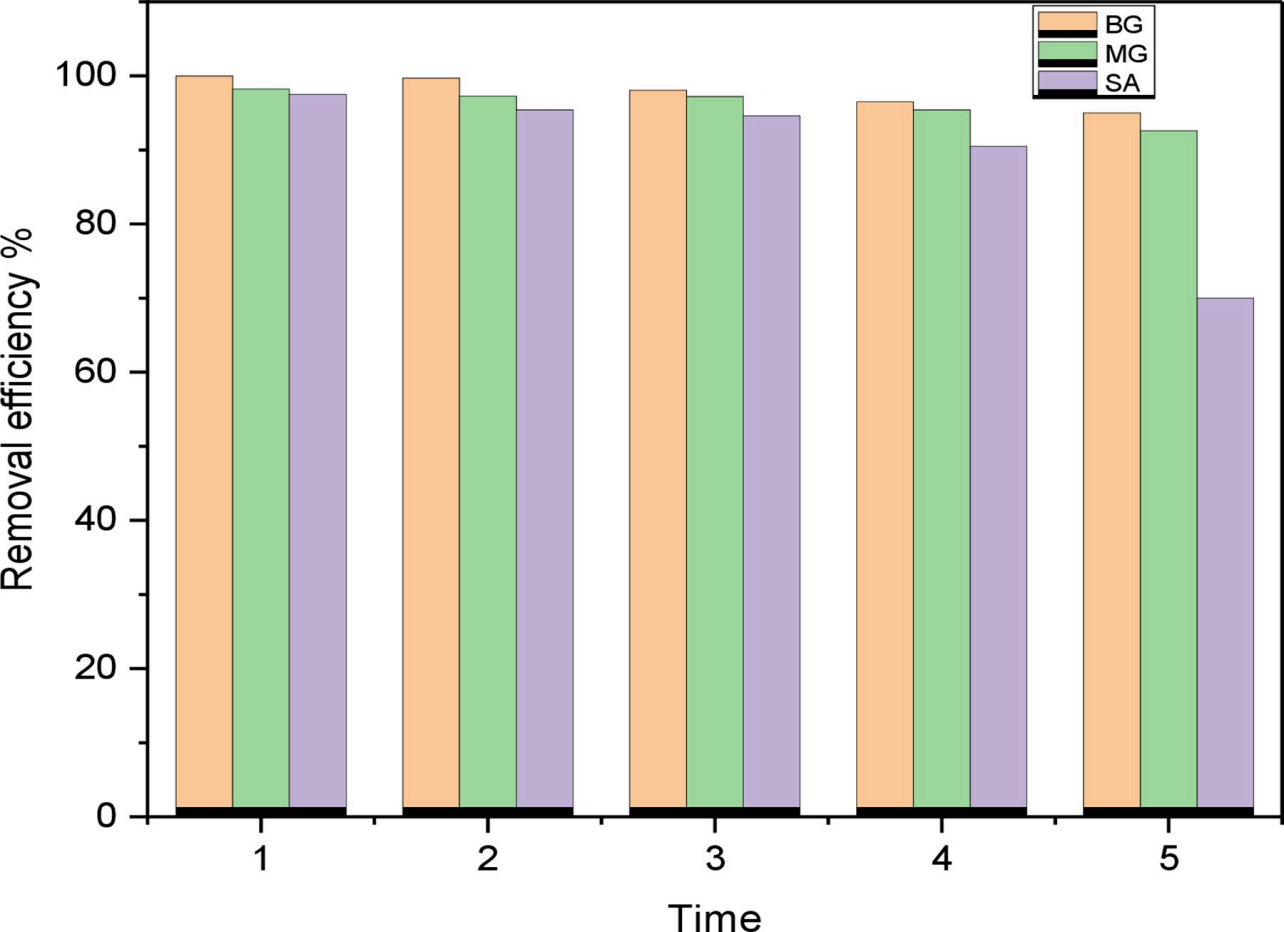
Reusability of adsorbent for dyes removal percent.

Regeneration and reusability of valuable adsorbates is an important aspect of adsorption technology. Here, desorption of adsorbent was tested by evaluating the dyes adsorption efficiency through treatment with four different eluents (deionized distilled water, ethanol, methanol, and nitric acid); the best eluent is ethanol. After the desorption test, the adsorbent’s reusability was tested for up to five adsorption-desorption cycles for all dyes as shown in **Fig 10**. The bar chart show the relationship between the number of recycles and the removal efficiency of the regenerated adsorbent. It can be seen that the removal efficiency of the adsorbent remained constant and no significant change in removal efficiency was observed during the five adsorption-desorption cycles for BG and MG. These data are encouraging and propose that the adsorbent in this experimental study has good reusability. While for SA dye molecule after the third adsorption-desorption cycle the removal efficiency decreased gradually and stayed at 70% for five cycles.

#### The chemical interaction- mechanical suggestion

A suggested adsorption mechanism between target dye molecules and functional groups of crown ether as shown in **S5 Fig**. As can be seen, electrostatic attraction and dipole–dipole interactions are found to be the dominant interactions between target dye and adsorbent surface. At higher solution pH values, the surface of crown ether particles become a negatively charge (-OH group) and the electropositive groups (nitrogen (N) atom) on the cationic dye molecules, As a result, dye ions are thus more favorably adsorbed onto the surfaces of the adsorbent at relative higher pH values. FESEM and EDX give another support for this mechanism (host-guest) by occupied all the pores in the adsorbent surface and present of dye atoms in EDX chart. A similar mechanism has been previously reported by Fayazi M. et al [39].

#### Comparison with other adsorbents

BG, MG, and SA adsorption capacity were considerable compared to other adsorbents found in the literature, as shown in **Table 4**.

**Table 4:** Comparison of BG, MG, and SA adsorbent in different adsorbent materials.

Adsorbents	Dyes	Adsorption capacity (mg/g)	References
**Mn0.5Cu0.5Fe_2_O_4_ nanospinels**	BG	0.89	[48]
**Rambutan peels**		9.64	[49]
**Tannin gel (TG)** **mine modified tannin gel (ATG)**		20.41 8.55	[50]
**Salix alba leaves (SAL)**		15.89	[51]
**MC (HOTTD)**		19.26	This study
**Treated sawdust**	MG	21.7	[52]
**Rice husk treated with NaOH**		12.16	[53]
**ZnO-NR-AC**		20	[54]
**Activated carbon (AC)**		28.5	[55]
**MC (HOTTD)**		18.28	This study
**Rice husk (treated with NaOH)**	SA	9.77	[56]
**Kaolinite (NRK) Clay**		16.23	[57]
**Iron oxide/sepiolite**		18.48	[39]
**Bambusa tulda**		32.26	[58]
**Nano FeO/CA**		1.910	[59]
**MC (HOTTD)**		14.35	This study

From Table 4, various adsorbents have been used for uptake of BG, MG and SA. Each bio sorbent has different adsorption capacity depends on the adsorbent nature and functional groups. The new prepared crown ether show high removal capacity during 10 min at room temperature and pH 9.0 for both BG and MG while 70 min for SA.

## Conclusions

The effectiveness of a novel macrocyclic molecule as an adsorbent for removing BG, MG, and S dye was proven. The effect of adsorbent dose, contact duration, and solution pH on dye absorption was determined. The following parameters were found to be optimal after optimizing the variables indicated above: 50 mg of adsorbent, contact time of 10 minutes for both BG and MG, and 70 minutes for SA, and pH >8.0. A high percentage of dye removal was observed based on this improved process. The equilibrium adsorption data revealed that both models, pseudo-second-order, and Langmuir, very effectively described the dyes adsorption process of the adsorbent. According to the Langmuir model, the maximum adsorption capacity (q_m_) for BG, MG, and SA was 19.29, 18.28, and 14.35 mg/g, respectively. Overall, this research suggests that the synthesized adsorbent has a high potential for usage in removing dyes in wastewater treatment.

## Supporting information

S1 Fig ^1^H-NMR spectrum of compound (1). (DOCX)

S2 Fig ^1^H-NMR spectrum of compound (2). (DOCX)

S3 Fig ^1^H-NMR spectrum of compound (3). (DOCX)

S4 Fig (a) pseudo-first-order kinetics model, (b) pseudo-second-order kinetics model, and (c) Intra-particle diffusion kinetic model for adsorption of BG, MG, and SA by MC adsorbent.
(DOCX)

S5 Fig Possible Safranin (SA) adsorption mechanism on the adsorbent structure. (DOCX)
