# Supplementary figures and images for: Removal of dyes (BG, MG, and SA) from aqueous solution using a novel adsorbent macrocyclic compound
Source: PLoS One. 2022 Oct 6;17(10):e0275330. doi: 10.1371/journal.pone.0275330 (PMC9536618; doi:10.1371/journal.pone.0275330)

Fig S1 .1H-NMR spectrum of compound (1).


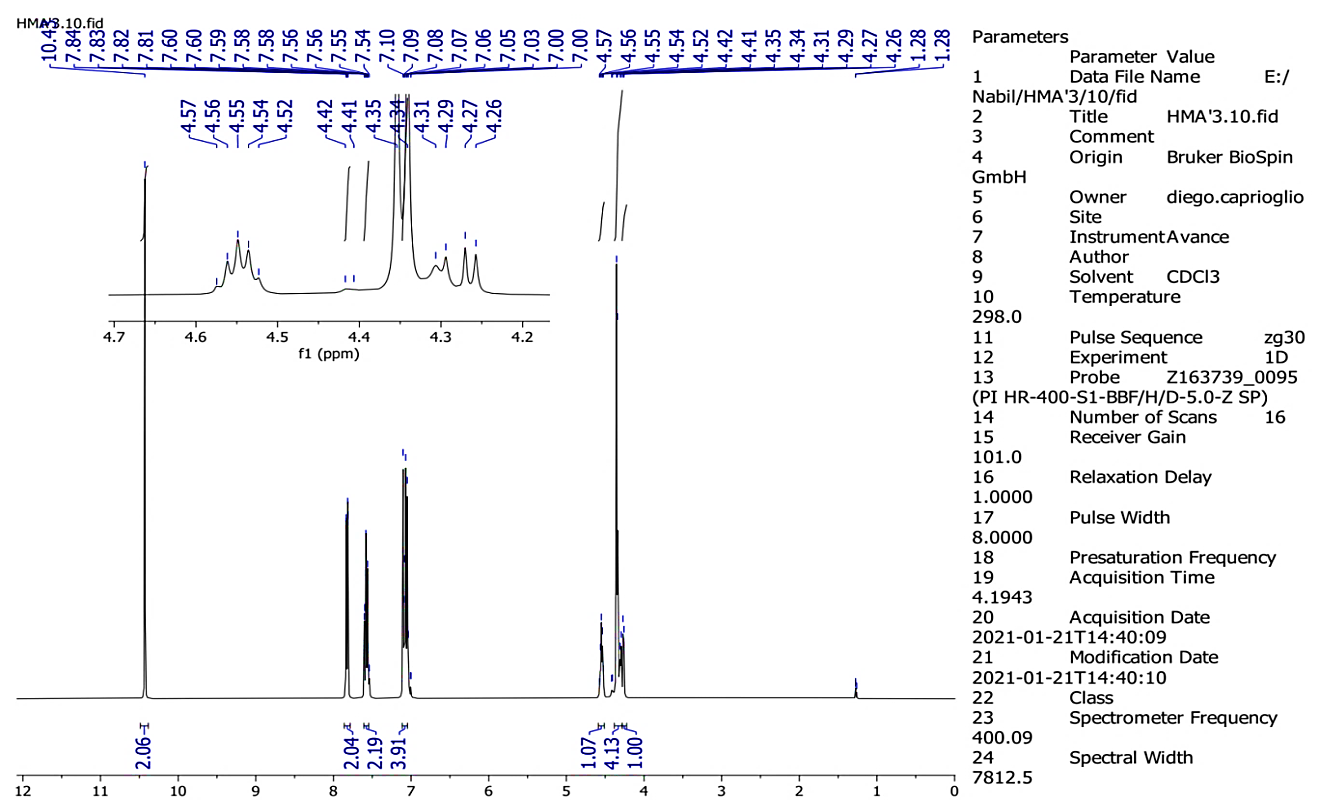

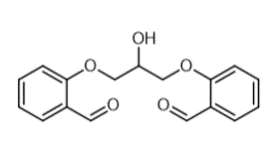

Supplement: S1 Fig — (DOCX) [file pone.0275330.s001.docx]

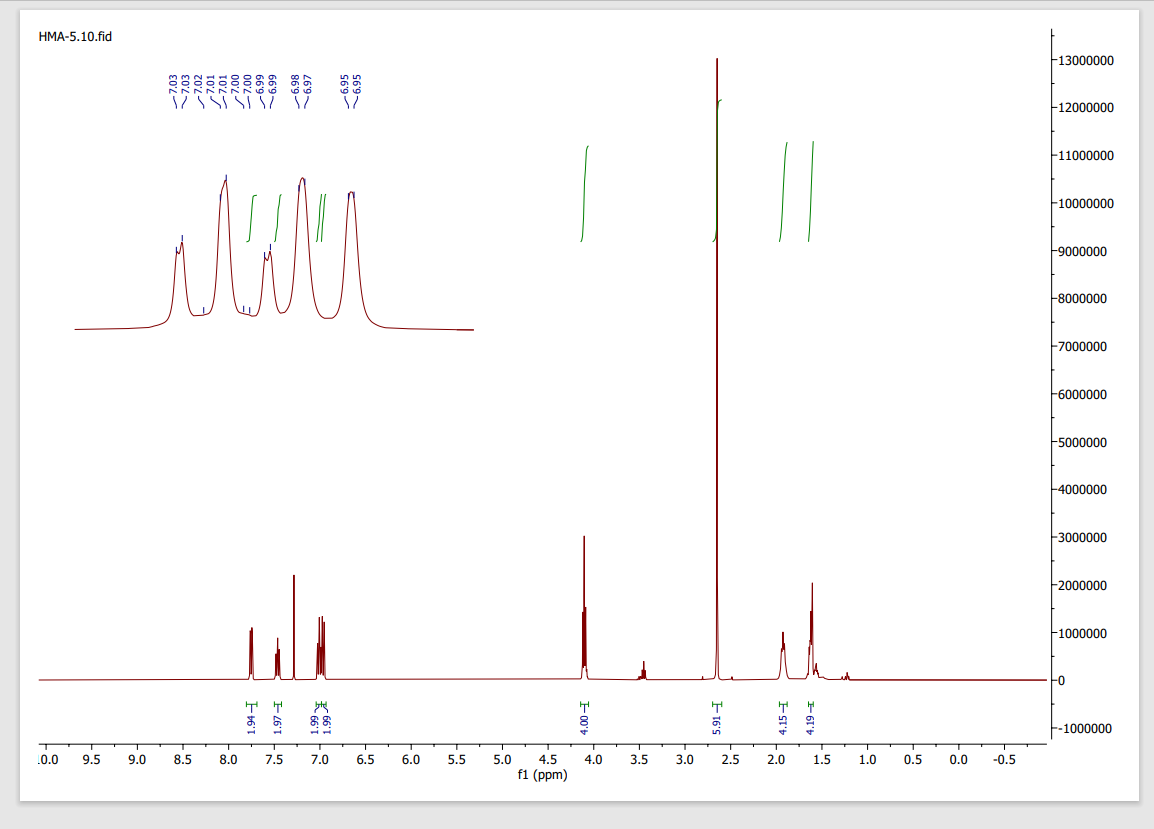


Fig S2 1H-NMR spectrum of compound (2).

Supplement: S2 Fig — (DOCX) [file pone.0275330.s002.docx]

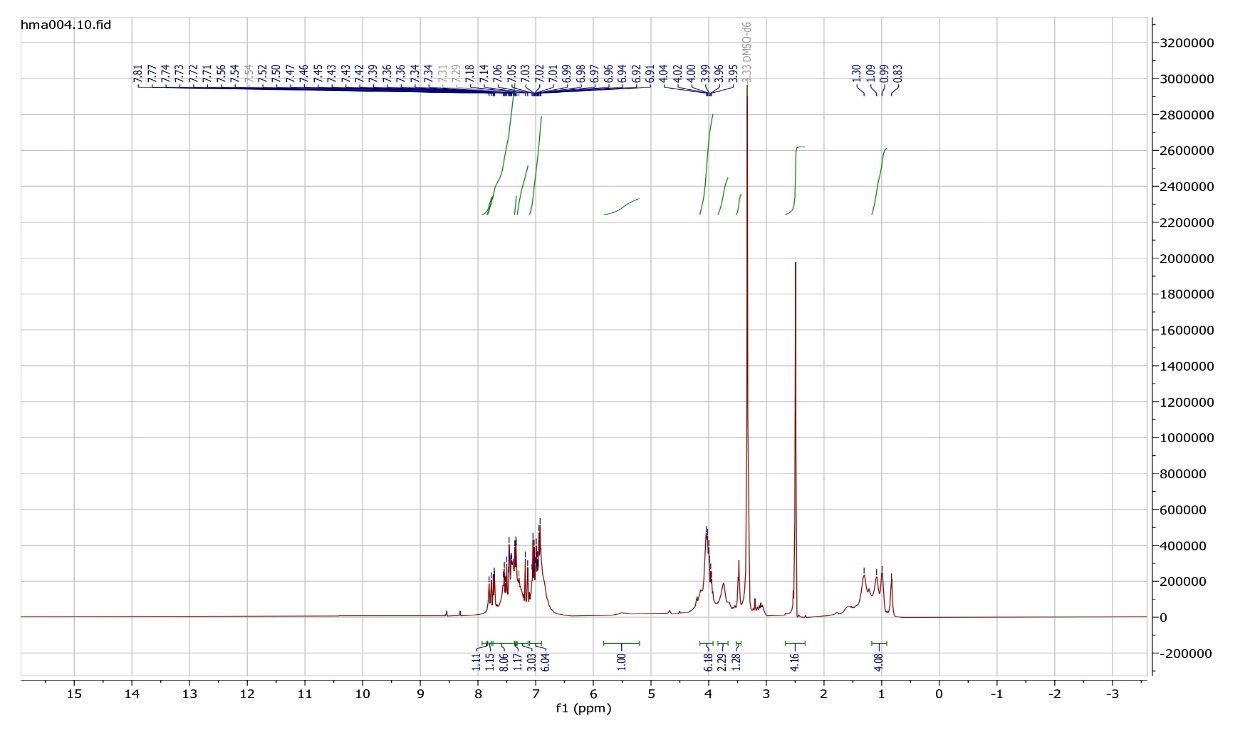


Fig S3 1H-NMR spectrum of compound (3).

Supplement: S3 Fig — (DOCX) [file pone.0275330.s003.docx]

**
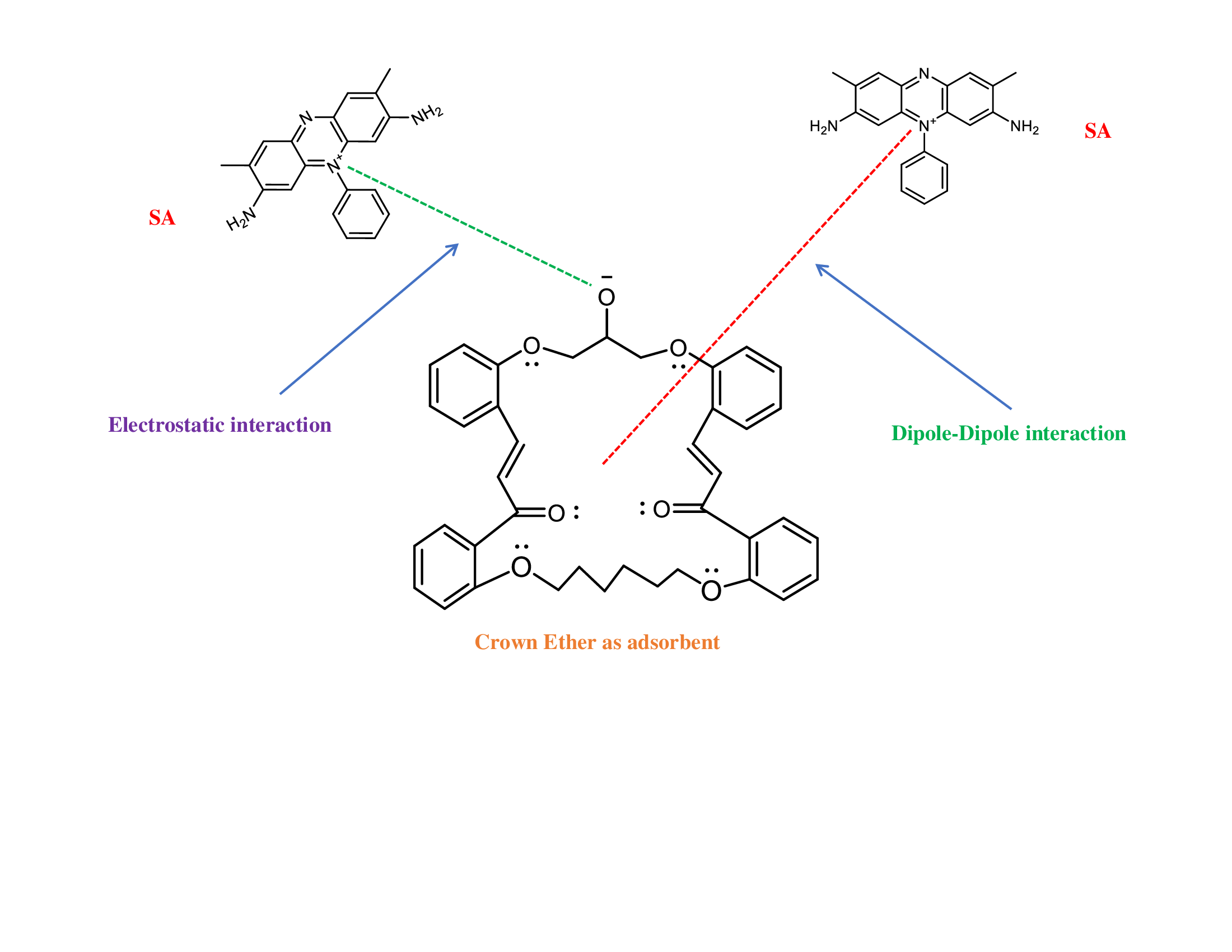
**

**Fig S5 Possible Safranin (SA) adsorption mechanism on the adsorbent structure.**

Supplement: S5 Fig — (DOCX) [file pone.0275330.s005.docx]
