# Supplementary material for: Removal of dyes (BG, MG, and SA) from aqueous solution using a novel adsorbent macrocyclic compound
Source: PLoS One. 2022 Oct 6;17(10):e0275330. doi: 10.1371/journal.pone.0275330 (PMC9536618; doi:10.1371/journal.pone.0275330)
Supplement: S4 Fig — (a) pseudo-first-order kinetics model, (b) pseudo-second-order kinetics model, and (c) Intra-particle diffusion kinetic model for adsorption of BG, MG, and SA by MC adsorbent. (DOCX) [file pone.0275330.s004.docx]

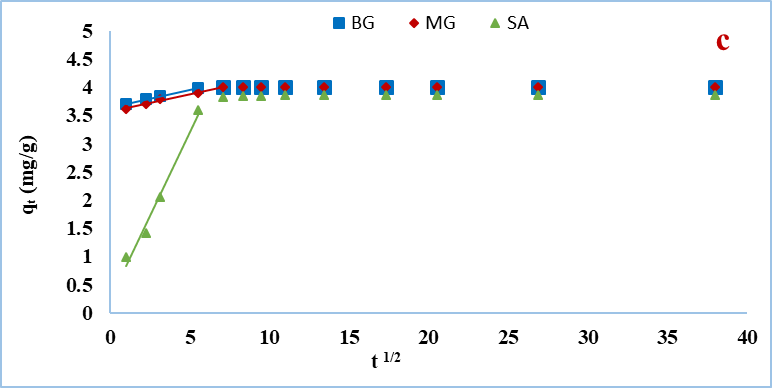

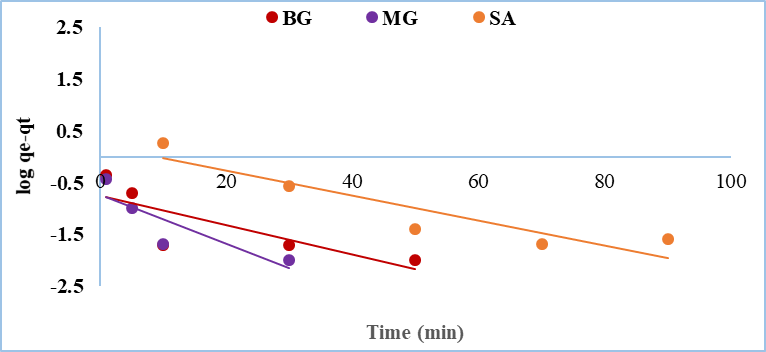

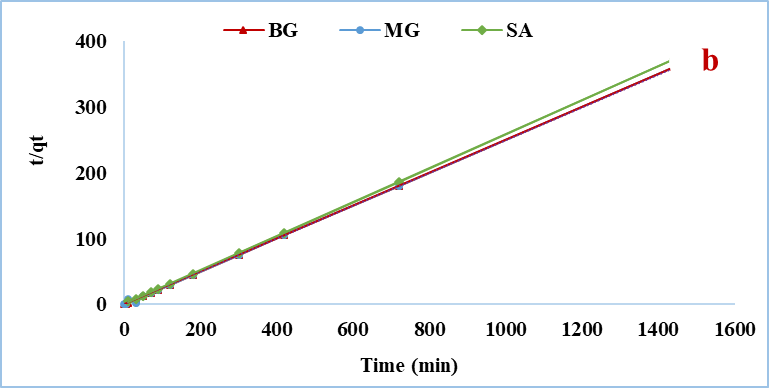


**Fig S4 (a) pseudo-first-order kinetics model, (b) pseudo-second-order kinetics model, and (c) Intra-particle diffusion kinetic model for adsorption of BG, MG, and SA by MC adsorbent**
